# Content validity and measurement properties of the Lower Extremity Functional Scale in patients with fractures of the lower extremities: a systematic review

**DOI:** 10.1186/s41687-022-00417-2

**Published:** 2022-01-29

**Authors:** Julia Ratter, Sylvia Pellekooren, Suzanne Wiertsema, Johanna M. van Dongen, Edwin Geleijn, Vincent de Groot, Frank W. Bloemers, Elise Jansma, Raymond W. J. G. Ostelo

**Affiliations:** 1 Department of Rehabilitation Medicine, Amsterdam Movement Sciences, Amsterdam UMC, Vrije Universiteit Amsterdam, De Boelelaan 1117, 1081 HZ Amsterdam, The Netherlands; 2 Department of Health Sciences, Faculty of Science, Amsterdam Movement Sciences, Vrije Universiteit Amsterdam, Amsterdam, The Netherlands; 3 Department Human Movement Sciences, Faculty of Behavioral and Movement Sciences, Vrije Universiteit Amsterdam, De Boelelaan 1117, Amsterdam, The Netherlands; 4 Department of Trauma Surgery, Amsterdam Movement Sciences, Amsterdam UMC, Vrije Universiteit Amsterdam, De Boelelaan 1117, Amsterdam, The Netherlands; 5 Department of Epidemiology and Data Science, Amsterdam Public Health Research Institute, Amsterdam UMC, Vrije Universiteit Amsterdam, Amsterdam, The Netherlands; 6 Department of Epidemiology and Data Science, Location VUmc, Amsterdam Movement Sciences, Amsterdam UMC, De Boelelaan 1117, Amsterdam, The Netherlands

**Keywords:** Content validity, Measurement properties, Lower Extremity Functional Scale, Fracture(s), Review (publication type), COSMIN

## Abstract

**Background:**

Fractures of lower extremities are common trauma-related injuries, and have major impact on patients' functional status. A frequently used Patient-Reported Outcome Measure (PROM) to evaluate patients’ functional status with lower extremity fractures is the Lower Extremity Functional Scale (LEFS). However, there is no systematic review regarding content validity and other measurement properties of the LEFS in patients with lower extremity fractures.

**Methods:**

A search was performed in PubMed, Embase, Scopus, and Cochrane Library from inception until November 2020. Studies on development of the LEFS and/or the evaluation of one or more measurement properties of the LEFS in patients with lower extremity fractures were included, and independently assessed by two reviewers using COSMIN guidelines.

**Results:**

Seven studies were included. Content validity of the LEFS was rated 'inconsistent', supported by very low quality of evidence. Structural validity was rated ‘insufficient’ supported by doubtful methodological quality. Internal consistency, measurement error, and responsiveness were rated 'indeterminate' supported by inadequate to adequate methodological quality. The methodological quality of the construct validity (hypotheses testing) assessment was rated as 'inadequate'.

**Conclusion:**

The LEFS has several shortcomings, the lack of sufficient content validity being the most important one as content validity is considered the most crucial measurement property of a PROM according to the COSMIN guidelines. In interpreting the outcomes, one should therefore be aware that not all relevant aspects of physical functioning may be accounted for in the LEFS. Further validation in a well-designed content validity study is needed, including a clearly defined construct and patient involvement during the assessment of different aspects of content validity.

**Plain English summary:**

Bone fractures of the lower extremities are a common injury. During rehabilitation it is essential to evaluate how patients experience their physical functioning, in order to monitor the progress and to optimize treatment. To measure physical functioning often questionnaires (also known as Patient Reported Outcome Measures) are used, such as the Lower Extremity Functional Scale (LEFS). However, it is not clear if the LEFS actually measures physical function, and if its other measurement properties are sufficient for using this questionnaire among patients with fractures in the lower extremities. Therefore, we systematically searched and assessed scientific papers on the development of the LEFS (i.e., its ability to measure physical functioning), and papers on the performance of the LEFS with regard to several measurement properties to identify possible factors that may cause measurement errors. Hereby we have assessed the quality of the studies included. Our main finding was that the LEFS may not measure all aspects of physical function. Given the low quality of the papers included in our study, these findings come with considerable uncertainty. As the LEFS was developed more than 20 years ago, it may not represent physical functioning as we currently conceptualize this. Therefore, we recommend to perform a study in which the content of the LEFS will be evaluated by experts in the field as well as patients, and modify the questionnaire as needed.

## Background

Fractures of the lower extremities are a common injury. Moreover, as life expectancy is generally increasing and the risk of osteoporotic fractures typically grows with age, lower extremity fractures are a rising source of morbidity, particularly in the elderly population [1–3]. In younger patients, fractures are more frequently sustained from high-energy or sports-related trauma [4–6]. Although data on the worldwide incidence of fractures are scarce and oftentimes outdated, studies suggest that their worldwide incidence ranges from 9.0 to 22.8 fractures per 1000 person-years [7, 8], and fractures of the lower limb account for approximately one third of all fractures [9–11].

Fractures of the lower extremities have a major impact on patients' functional status [5, 10, 12–14]. Due to a variation of types of injury and treatment and the variation in the natural recovery process of traumatic fractures patients with fractures typically differ from patients with other lower extremity dysfunction, for instance rheumatism.

After traumatic injury, maximizing patients' recovery relies heavily on optimizing their functional status and minimizing their symptoms [15–17]. Using a validated Patient-Reported Outcome Measure (PROM) helps identify and address these outcomes in clinical practice [18, 19]. PROMs are designed to quantify the patients' health, health-related quality of life, or functional status without interpretation of the patients' response by a clinician [14, 20–22].

A frequently used PROM to examine the functional status of patients with lower extremity fractures is the Lower Extremity Functional Scale (LEFS) [23, 24]. The LEFS is a self-administered questionnaire containing 20 questions about a person's ability to perform everyday tasks. The scale ranges from 0 to 80, with higher scores indicating better function.

Two systematic reviews have assessed the measurement properties of the LEFS [24, 25]. Although these systematic reviews concluded that the LEFS had good reliability, validity, and responsiveness [24, 25], no comprehensive assessment on content validity was performed, and none of these studies focused on the measurement properties of the LEFS in patients with fractures of the lower extremities in particular [26]. Therefore, this study aimed to systematically review the literature to evaluate the content validity and other measurement properties of the LEFS in patients with fractures of the lower extremities in accordance with the COSMIN methodology for systematic reviews of Patient‐Reported Outcome Measures (PROMs) [26].

## Methods

This review was conducted in accordance with the COSMIN methodology for systematic reviews of Patient‐Reported Outcome Measures (PROMs) [26]. A protocol was written a priori and was registered prospectively in PROSPERO (registration number: CRD42020184557).

### Data sources and study selection

A search was performed in PubMed (including Medline), Embase, Scopus, and the Cochrane Library from inception until November 2020. The initial search was conducted together with an experienced clinical librarian (EJ) on 27 May, and updated on 3 November 2020. The search strategies are presented in Appendix 1. Additionally, a forward citation search was performed in Google Scholar, and references of included studies were cross-checked.

Eligible studies had to report on the development of the LEFS or the evaluation of one or more measurement properties of the LEFS in patients with at least one fracture of the lower extremities. As content validity is considered the most crucial measurement property of a PROM [27], we decided to include the original development study of the LEFS, irrespective of the study population, which is in line with the COSMIN methodology for systematic reviews of Patient‐Reported Outcome Measures (PROMs) [26]. According to the guideline of Prinsen et al. [28] ‘content validity is defined as ‘the degree to which the content of an instrument is an adequate reflection of the construct to be measured’ is the first measurement property that should be assessed when selecting an instrument, as it allows making a link between the content of the instrument and that of the construct to be measured.’

Studies reporting on all other measurement properties had to have a study sample consisting largely of patients with at least one fracture of the lower extremity (≥ 75% of the sample) [26].

No timing criteria for the fractures of the lower extremities were used as inclusion criteria. Studies published in any language were eligible for inclusion, in accordance with the COSMIN methodology for systematic reviews of Patient‐Reported Outcome Measures (PROMs) [26]. Studies that used the LEFS as an outcome measure or studies that used the LEFS to assess another instrument's measurement properties were excluded [26].

Records retrieved by the search were independently assessed for eligibility by two reviewers (JR, SP). The initial selection was based on title and abstract. Potentially eligible studies were assessed by obtaining the full-text to confirm eligibility. Discrepancies between reviewers were reviewed, and consensus was achieved by discussion.

### Data extraction and quality assessment

Data on the characteristics of the study population (i.e., sample size, age, gender, proportion of total sample consisting of fracture patients, location fracture, treatment, time since fracture/treatment) and instrument administration (i.e., setting, country, language) were extracted by one reviewer (JR) and checked by a second reviewer (SP). A customized data extraction form was developed for this purpose, based on the COSMIN guidelines [26]. The methodological quality of the included studies was assessed by two independent reviewers (JR, SP), using the COSMIN Risk of Bias (RoB) checklist [26].

This checklist included ten separate boxes with standards for individual assessment of PROM development (box 1), and for nine measurement properties (box 2- 10) according to the COSMIN taxonomy which is based on the COSMIN guidelines [26]. The order and structure of evaluating the measurement properties were in line with the COSMIN methodology for systematic reviews of Patient‐Reported Outcome Measures (PROMs) [26], i.e.:

- *Content validity*: PROM development (not a measurement property, but taken into account when evaluating content validity) and content validity;
- *Internal structure*: structural validity, internal consistency, Cross‐cultural validity/ measurement invariance;
- *Remaining measurement properties*: reliability, measurement error, criterion validity, hypotheses testing for construct validity, responsiveness [29].

In our protocol we had included the evaluation of all measurement properties. However, none of the included studies evaluated cross-cultural validity and criterion validity and therefore these measurement properties were not further evaluated.

The assessment of *content validity* required slightly different steps than assessing *internal structure* and the *remaining measurement properties*, both of which will be discussed in more detail below.

To assess the LEFS’s *content validity,* the COSMIN guideline for systematic reviews of PROMs [26] as well as an additional guideline for evaluating the content validity of PROMs were used [27], and the three following steps were conducted:

1. Evaluation of the quality of the PROM development: The quality of the PROM development was evaluated by two independent reviewers (JR, SP), using the COSMIN Risk of Bias checklist box 1, which consists of two parts (quality of the PROM design, quality of a cognitive interview study or other pilot test).
2. Evaluation of the quality of all additional content validity studies on the PROM (if available): If available, the quality of additional content validity studies was evaluated using the COSMIN Risk of Bias checklist box 2, concerning relevance, comprehensiveness, and comprehensibility of the PROM.
3. Evaluation of the content validity of the PROM, based on the quality and results of the available studies and the PROM itself against the ten criteria for good content validity: In this step, the content validity of the PROM was rated by two independent reviewers (JR, SP), based on a summary of all available evidence on the PROM development and additional content validity studies, if available. In addition, according to the COSMIN guideline [27], the reviewers rated the content of the PROM themselves hereby using additional literature linking ICF categories on to the LEFS [30].

To assess the LEFS’s *internal structure* and the *remaining measurement properties,* the three following steps were conducted:

1. Methodological quality assessment: The methodological quality of the included studies was assessed by two independent reviewers (JR, SP), using the COSMIN Risk of Bias (RoB) checklist [26]. The studies’ methodological quality was assessed per measurement property separately. That is, per measurement property, only the boxes pertaining to that measurement property were used. Each box consists of four or more items, all of which were rated on a 4-point rating scale (i.e., “very good”, “adequate”, “doubtful”, or “inadequate”). The studies' overall score per measurement property was equal to the lowest rated item of the respective box (i.e., "the worst score counts" principle). Discrepancies between reviewers were discussed and solved by consensus.
2. Measurement property assessment: The results of every single study on a specific measurement property (e.g., ICC or weighted Kappa) were extracted and subsequently rated according to the updated criteria for good measurement properties as being “sufficient”, “insufficient” or “indeterminate”[26], as stated in the COSMIN guideline [26].
3. Summarizing and grading the evidence: In our protocol we had included “quantitatively pooling of the results” and “grading the evidence of all available studies in accordance with the GRADE approach”. However, based on the included studies, we were not able to perform these steps due to insufficient homogeneity in both statistical analysis and study population, and the inconsistency of results of all available studies per measurement property [26].

## Results

### Identified studies

**Fig. 1:**
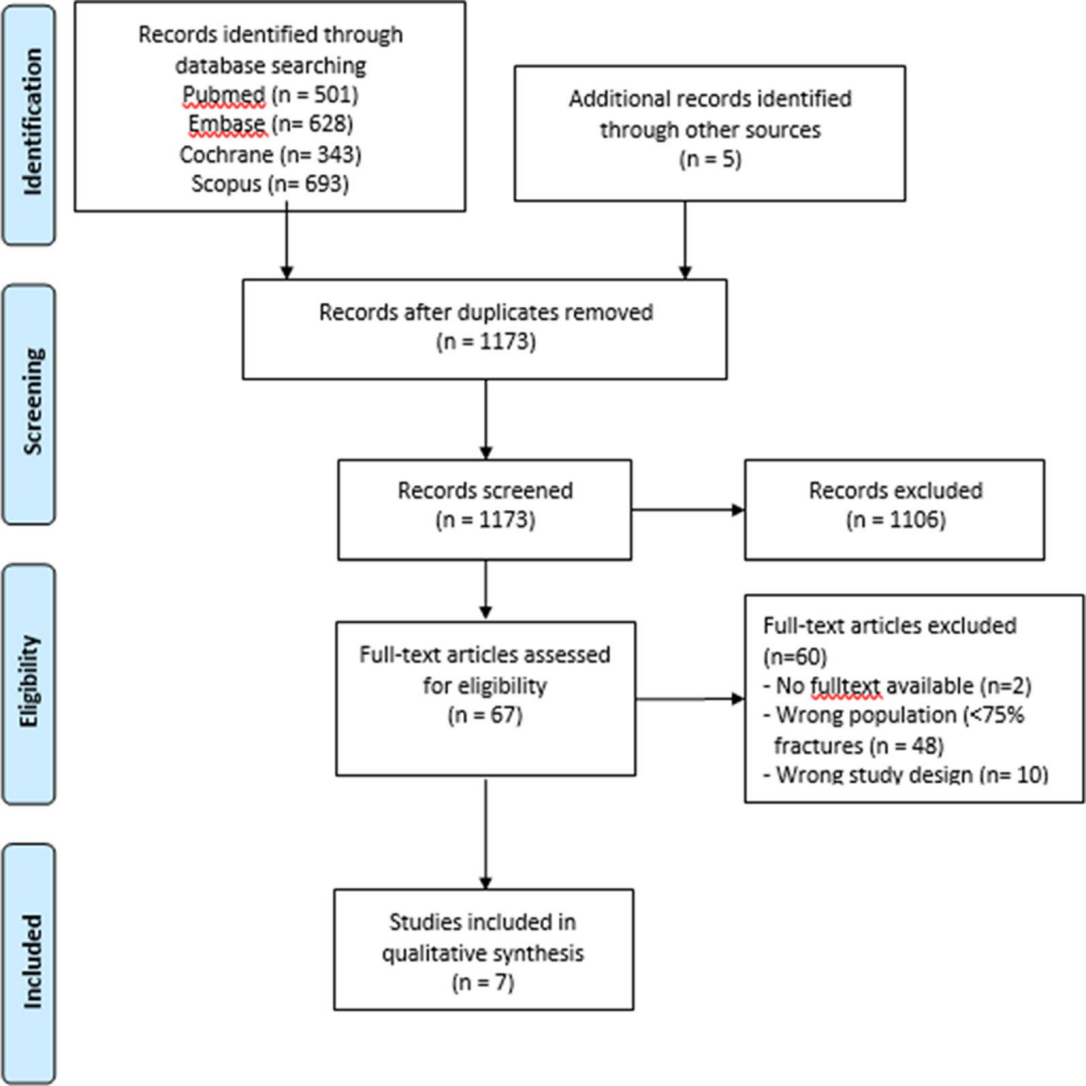
PRISMA flow diagram

The search yielded 2,170 records, equaling 1173 potentially relevant studies after removing duplicates. After initial screening, 67 full texts were obtained. The final selection included seven studies. Reasons for excluding studies included were: no full-text available (n = 2), wrong study population (e.g. musculoskeletal disorders) (n = 48) and wrong study design (e.g. studies that used the LEFS as an outcome measure or studies that used the LEFS to assess another instrument's measurement properties) (n = 10). More details of the search are presented in Fig. 1.

### Study characteristics

Sample sizes of the included studies varied from 20 [31] to 567 patients [32]. The mean age of the patients ranged from 38.0 [31] to 57.5 years [32], and 50.3% [33] to 70.0% [31] of the patients were female. These figures are based on the descriptive statistics where we rely on the reported numbers as published in the included studies. The setting in which the measurement properties of the LEFS were assessed differed between studies and included a physical therapy clinic [23], a (teaching) hospital [32, 34, 35], a rehabilitation department [31, 33], and records from a national electronic database on post-operative patients [36]. The LEFS was assessed in four languages, including English [23, 33], Norwegian [32], Chinese [31], and Finnish [34–36]. All included studies met the criterion of having at least 75% subjects with a fracture of the lower extremity, except for Binkley et al.'s [23] development study, where only 10.2% had a lower extremity fracture. Furthermore, Hsu et al. [31] included patients with ankle fractures and a group of age- and sex-matched healthy controls. This study was included because more than 75% of the fracture patient group had a fracture of the lower extremities. The LEFS was administered directly after (surgical) treatment [23] until several years after trauma [31–36]. Fractures were located in different lower extremities regions, mostly the ankle/foot region [23, 31–36]. More details on the characteristics of the studies are presented in Table 1.

**Table 1:** Characteristics of the studies

	Population				Fracture characteristics			Instrument administration		
Studies in alphabethic order	N	Age Mean (SD, range) yr	Gender % female	Fracture (% of total sample)	Location fracture(s)	Treatment surgical/ non-surgical	Time since fracture/treatment	Setting	Country	Language
Binkley, Stratford [23]	107	Mean 44 (SD 16.2)	54.2%	10.2%	Knee, thigh, foot, ankle	Unclear	6 (0–250) weeks since onset	Physical therapy clinics	United States and Canada	English
Garratt, Naumann [32]	959 (returned: 567)	Mean 57.5 (Range 22.2- 91.2)	56.8%	100%	Ankle (Weber A 2.6%, Weber B 67.5%, Weber C 27.5%)	Operative fixation of closed ankle fractures	> 3 years after treatment	2 hospitals in SE Norway	Norway	Norwegian
Hsu, Tsai [31]	20	Median 38.0 (IQR 18.0)	70%	50% (100% cases)	Ankle	Cast Immobilization ± open reduction and internal fixation	4 months (median) after fracture	Rehabilitation department of a teaching hospital	Taiwan	Chinese
Lin, Moseley [33]	306	Mean 45.1 (SD 15.7)	50.3%	100%	Ankle	56.3% surgical	Within 7 days of cast removal	Outpatient physical therapy departments of 3 teaching hospitals, outpatient orthopedic clinic of 2 teaching hospitals	Australia	English
Ponkilainen, Tukiainen [36]	165	Mean 54.6 (SD 19.7)	54.5%	95.5% trauma (n = 156), infection (n = 6), tumor (n = 2) or osteoarthritis (n = 1)	Ankle (n = 133), hindfoot (n = 16), midfoot (n = 7), forefoot (n = 3) or more locations (n = 6)	Ankle fracture osteosynthesis, removal of implants from foot or ankle, tibiotalar joint fusion	Averagely 4 years (range from 1 month to 10 years) after surgery	Database	Finland	Finnish
Repo, Tukiainen [34]	166	Mean 55.0 (SD 16.0)	53%	90%	ankle, foot	100% surgical	4 years (0–14) time since surgery	Helsinki University Hospital	Finland	Finnish
Repo, Tukiainen [35]	182	Mean 55.0 (SD 16.0)	53.5%	89%	ankle, foot	100% surgical	3.2 years (9.6) since surgery	Helsinki University Hospital	Finland	Finnish

Seven studies were included, including one study that evaluated the development of the LEFS [23]. No additional content validity studies were identified. Five studies [32–36] evaluated structural validity, four studies [32–35] evaluated internal consistency, two studies [32, 34] evaluated reliability, two studies [32, 35] evaluated measurement error, and three studies [31, 32, 34] evaluated construct validity (i.e. hypotheses testing). One study [33] evaluated two aspects of responsiveness (i.e. hypotheses testing: comparison with other outcome measurement and hypotheses testing: before and after intervention). None of the studies evaluated cross-cultural validity and criterion validity and therefore were not further evaluated.

### Methodological quality and measurement property assessment

#### PROM development and content validity

One study was identified on the development and initial assessment of the LEFS [23], whereas no additional studies were identified on the content validity of the LEFS. A clear description of the construct that the LEFS sets out to measure was missing from the identified development study, and the LEFS’ conceptual framework was unclear. Moreover, no cognitive interview or pilot test was performed in which patients were asked about the comprehensiveness and comprehensibility of the LEFS. Therefore, all of these items were scored as ‘inadequate’. As the PROM development's overall methodological quality was rated 'inadequate' an ‘indeterminate’ rating was given for relevance, comprehensiveness and comprehensibility.

In accordance with the COSMIN guidelines, the content validity of the LEFS was then rated subjectively by the reviewers [26]. Reviewers rated both relevance and comprehensibility as 'sufficient' and comprehensiveness as 'inconsistent'. The latter was due to the fact that reviewers found that probably not all key concepts regarding patients with fractures of the lower extremities were included in the development of the LEFS. ICF categories *d4 mobility* (e.g. movement with equipment and using transportation such as a bike or public transport) and *d5 self-care* (e.g. toileting and caring for body parts) may not be sufficiently covered. Hence, the LEFS’ content validity was 'inconsistent', supported by a very low level of evidence. The rating of the PROM development study's results against the ten criteria for good content validity is provided in Table 2.

**Table 2:** Content validity assessment

	Development study	Rating of reviewers	OVERALL RATINGS	QUALITY OF EVIDENCE	
*Score:* + = *sufficient;—*= *insufficient; ?* = *indeterminate;* ± = *inconsistent*	** ± / ± / ?**	** ± / ± / ?**	** ± / ± / ?**	**High, moderate, low, very low**	
	**consensus**	**consensus**	**consensus**		
**Relevance**					
1	Are the included items relevant for the construct of interest?^1^	?	+		
2	Are the included items relevant for the target population of interest?^1^	?	+		
3	Are the included items relevant for the context of use of interest?^1^	?	+		
4	Are the response options appropriate?	?	+		
5	Is the recall period appropriate?	?	+		
	**RELEVANCE RATING (± / ± / ?)**	**?**	** + **	** + **	
**Comprehensiveness**					
6	Are all key concepts included?	?	** ± **		
	**COMPREHENSIVENESS RATING (± / ± / ?)**	**?**	** ± **	** ± **	
**Comprehensibility**					
7	Are the PROM instructions understood by the population of interest as intended?	?			
8	Are the PROM items and response options understood by the population of interest as intended?	?			
9	Are the PROM items appropriately worded?		+		
10	Do the response options match the question?		+		
	**COMPREHENSIBILITY RATING (± / ± / ?)**	**?**	** + **	** + **	
	**CONTENT VALIDITY RATING (± / ± / ?)**	**?**	** ± **	** ± **	**Very low**

^1^ These criteria refer to the construct, population, and context of use of interest in the systematic review

#### Structural validity

In accordance with the COSMIN methodology for systematic reviews of Patient‐Reported Outcome Measures (PROMs) ‘structural validity conceptualizes the degree to which the scores of a PROM are an adequate reflection of the dimensionality of the construct to be measured ‘[26].

Five studies [32–36] evaluated the structural validity of the LEFS. The methodological quality of the structural validity assessment was rated as 'doubtful' in four of these studies [32–34, 36]. This was mainly due to insufficient reporting. The remaining study [35] was rated 'adequate'. The assessment of the methodological quality of the included studies using the COSMIN RoB checklist is provided in Table 3. Studies that included classical test theory (CTT) were assessed based on the use and outcomes of the comparative fit index (CFI) or Tucker‐Lewis index (TLI). Studies that included IRT/Rasch analyses were assessed bases on the assumptions of no violation of unidimensionality, local independence and monotonicity, and an adequate model fit. One study [36] found the LEFS to measure a unidimensional construct, based on “principal component (PC) analysis”. Four studies [32–35] found it to measure a multidimensional construct, based on “TLI”[32], “IRT”[33, 35], respectively “maximum likelihood factor analysis with oblimin rotation”[34]. The structural validity is insufficient because the results of the different studies do not give a convincing picture of the unidimensionality of the LEFS. Therefore the structural validity of the LEFS was rated 'insufficient'. The rating of the results of every single study on a measurement property against the updated criteria for good measurement properties is provided in Table 3.

**Table 3:** Methodological quality and assessment measurement properties

	Structural validity		Internal consistency		Reliability	
	Risk of bias	Assessment measurement properties ^1^	Risk of bias	Assessment measurement properties	Risk op bias	Assessment measurement properties
Garratt, Naumann [32]	doubtful	CFA: LEFS unidimensional TLI = 0.99, RMSEA = 0.091; LEFS multidimensional TLI = 1.00, RMSEA 0.073 TLI waarde ( +), RMSAE waarde (?)	inadequate	Cronbach's alpha (scale) α = 0.96 sufficient structural validity (?), Cronbach's alpha ( +)	adequate	Test–retest ICC 0.91 ( +)
Hsu, Tsai [31]						
Lin, Moseley [33]	doubtful	IRT: criteria followed Z- standardized statistic less than 2.0 or ≥ 2.0 (-): 3 items failed to conform to Rasch expectations; no further information reported (?)	inadequate	Baseline Cronbach's alpha α = 0.92, short-term follow-up α = 0.94, medium-term follow-up α = 0.90 sufficient structural validity (?), Cronbach's alpha ( +)		
Ponkilainen, Tukiainen [36]	doubtful	Correlation LEFS scores with 15D total score 0.67 (0.57- 0.74), LEFS and FIT index 0.44 (0.31- 0.57), LEFS and VAS general health − 0.66 (− 0.76 to − 0.55) (?)				
Repo, Tukiainen [34]	doubtful	Maximum likelihood factor analysis with oblimin rotation: LEFS loads on two factors, no further information reported (?)	inadequate	Cronbach's alpha (scale) α = 0.96 sufficient structural validity (?), Cronbach's alpha ( +)	adequate	Test–retest ICC 0.93 (95% CI, 0.91- 0.95) ( +)
Repo, Tukiainen [35]	adequate	IRT: unideminsionality not violated ( +)	inadequate	Cronbachs alpha α = 0.95 sufficient structural validity (?), Cronbach's alpha ( +)		

	Measurement error		Hypotheses testing for construct validity*			
	Risk of bias	Assessment measurement properties	Risk of bias	assessment measurement properties		
Garratt, Naumann [32]	adequate	SDC individual 12.49, SDC group 0.93, MIC not defined (?)	inadequate	Spearman correlation LEFS and OMAS 0.86, LEFS and SEFAS − 0.84, LEFS and SF-36 physical function 0.85, LEFS and EQ-5D index 0.73, ASA classification − .026 *		
Hsu, Tsai [31]			inadequate	Correlation LEFS scores with walking speed (r = 0.60, *p* = 0.044) and step length (r = 0.68, *p* = 0.021) *		
Lin, Moseley [33]						
Ponkilainen, Tukiainen [36]						
Repo, Tukiainen [34]	adequate	SEM 4.1, MIC not defined (?)	inadequate	Spearman correlation LEFS and 15D total index r = 0.66, FIT index physical activity r = 0.46, LEFS and VAS Foot and ankle pain at rest r = − 0.5, LEFS and VAS Foot and ankle pain during activity r = − 0.69, LEFS and VAS Foot and ankle stiffness r = 0.62 *		
Repo, Tukiainen [35]						

	Responsiveness construct approach (i.e. hypotheses testing: comparison with other outcome measurement)		Responsiveness construct approach (i.e. hypotheses testing: before and after intervention)			
	Risk of bias	Assessment measurement properties	Risk of bias	Assessment measurement properties		
Garratt, Naumann [32]						
Hsu, Tsai [31]						
Lin, Moseley [33]	inadequate	Guyatt responsiveness ratio: short-term 1.99, medium-term 1.74; External criterion for improvement = the global perceived effect scale (?)	inadequate	Effect size short-term 1.92, medium-term 3.33; standardized response mean short-term 1.91, medium-term 2.95 (?)		
Ponkilainen, Tukiainen [36]						
Repo, Tukiainen [34]						
Repo, Tukiainen [35]						

^1^ “ + ” = sufficient,” – “ = insufficient, “?” = indeterminate

ASA classification = ASA (American Society of Anesthesiologists) physical status classification system

CFA = confirmatory factor analysis

IRT = item response theory

MIC = minimally important change

TFI = Tucker-Lewis Index

RMSEA = Root means square error of approximation

SDC = Smallest detectable change

OMAS = Olerund Molander Ankle Score

VAS = Visual analog scale

*Authors have decided not to rate this property due to unclear construct of the LEFS

#### Internal consistency

Internal consistency refers to “the degree of the interrelatedness among the items”[26]. The risk of bias in a study on internal consistency depends on the available evidence for structural validity because unidimensionality is a prerequisite for the interpretation of internal consistency analyses (i.e. Cronbach’s alpha’s). Therefore, the quality of evidence for internal consistency cannot be higher than the quality of evidence for structural validity [26]. Four studies [32–35] assessed the internal consistency of the LEFS. The methodological quality of all of these studies was rated 'inadequate'. The assessment of the methodological quality of the included studies using the COSMIN RoB checklist is provided in Table 3. The included studies calculated a Cronbach's alpha, all of which were 0.90[33] or higher [32, 34]. Even though this suggests that the items of the LEFS have relatively high internal consistency, the LEFS was found not to measure a unidimensional construct in one of the included studies [35]. The internal consistency of the LEFS was therefore rated as ‘indeterminate’ as outlined in the COSMIN guideline and was supported by three studies of lower methodological quality as well [32–34].

#### Reliability

Two studies [32, 34] assessed the test–retest reliability of the LEFS. The methodological quality of the reliability assessment in both included studies was rated as 'adequate'. The assessment of the methodological quality of the included studies using the COSMIN RoB checklist can be found in Table 3. The time interval between the first and the second measurement was on average 2.5 weeks [34], respectively six weeks [32]. Garratt [32] found the test–retest ICC of the LEFS to be 0.91, based on a two-way mixed effects model with absolute agreement. A weighted kappa was used for assessing individual item reliability [32]. Repo et al.[34] found a ICC of 0.93 (95% CI, 0.91- 0.95), based on a two-way mixed model with absolute agreement. Both of these ICCs indicate that the reliability of the LEFS is 'sufficient' (Table 3).

#### Measurement error

According to the COSMIN guideline, “measurement error refers to the systematic and random error of an individual patient’s score that is not attributed to true changes in the construct to be measured.”[26] When applying the criteria for good measurement error, information is needed on the Smallest Detectable Change (SDC) or Limits of Agreement (LoA), as well as on the Minimal Important Change (MIC) [26]. Two studies [32, 35] assessed the measurement error of the LEFS. The methodological quality of both of these two studies was rated as 'adequate'. The assessment of the methodological quality of the included studies using the COSMIN RoB checklist is provided in Table 3. Garratt et al. [32] found a smallest detectable change of 12.49. The minimal important change was not defined. Repo et al. [35] reported a Standard Error of Measurement of 4.1. In their study, the minimal important change was not defined. Consequently, the measurement error of the LEFS was rated as 'indeterminate' (Table 3).

#### Construct validity (hypotheses testing)

According to the COSMIN guideline, construct validity has 3 subsections, one of them being hypotheses testing. This refers to “the degree to which the scores of a PROM are consistent with hypotheses (for instance with regard to internal relationships, relationships to scores of other instruments, or differences between relevant groups) based on the assumption that the PROM validly measures the construct to be measured.”[26] According to the COSMIN guideline the risk of bias of studies comparing the PROM to comparison instruments was completed [26].

Three studies [31, 32, 34] evaluated the construct validity (hypotheses testing) of the LEFS. The methodological quality of the construct validity (hypotheses testing) assessment was rated as 'inadequate' for all included studies (Table 3). Due to an unclear definition of the construct the LEFS purports to measure, we did not further assess hypotheses testing for construct validity and did not apply criteria for good measurement properties.

### Responsiveness

Responsiveness refers to “the ability of a PROM to detect change over time in the construct to be measured”, according to the COSMIN guideline [26]. One study [33] evaluated two aspects of responsiveness (i.e. hypotheses testing: comparison with other outcome measurement and hypotheses testing: before and after intervention). The methodological quality of the responsiveness assessment was rated as 'inadequate' for the included study. The assessment of the methodological quality of the included study using the COSMIN RoB checklist can be found in Table 3. The responsiveness of the LEFS was rated as ‘indeterminate’ as outlined in the COSMIN guideline.

## Discussion

### Main findings

This study found the content validity of the LEFS to be 'inconsistent', which was supported by very low quality evidence. One study was identified on the development and initial assessment of the LEFS [23], whereas no additional studies were identified on the content validity of the LEFS. A clear description of the construct that the LEFS sets out to measure was missing from the identified development study, and the LEFS’ conceptual framework was unclear. Moreover, a study of ‘adequate’ methodological quality showed that the LEFS has a multidimensional construct [35]. The internal consistency of the LEFS was therefore rated as ‘indeterminate’ as outlined in the COSMIN guideline and was supported by three studies of lower methodological quality as well [32–34]. The reliability was rated ‘sufficient’[32, 34], based on two studies of adequate methodological quality. Measurement error was rated ‘indeterminate’[32, 34], based on two studies of adequate methodological quality. Responsiveness was rated ‘indeterminate’ [33], based on one study of inadequate methodological quality. Given the lack of clarity on the construct the LEFS aims to measure, hypotheses testing for construct validity was not assessed.

### Interpretation of the findings

As content validity is considered the most crucial measurement property of a PROM [27], it is of utmost importance that the construct a PROM sets out to measure, and the theoretical grounds which it is based on are clear. The development study of the LEFS did not include a clearly defined construct, and was based on an older version of the World Health Organization's model of disability and handicap [37], instead of the nowadays used more dynamic model of health in which health is defined as a process with a positive concept emphasizing social and personal resources, as well as physical capacities [38]. Therefore, the LEFS may not measure a patients’ physical functioning as we currently conceptualize this. Also, no appropriate cognitive interview was performed during the development or during additional validation studies, making it difficult to assess the relevance, comprehensiveness, and comprehensibility (e.g., ICF categories *d4 mobility* and *d5 self-care*) of the LEFS. For this reason, the LEFS encounters shortcomings regarding its content validity. We do acknowledge that the LEFS was developed many years before the COSMIN criteria, and the introduction of the dynamic model of health [34], however, we would like to endorse the fact that PROMS need to be fit for purpose when evaluating current health care. As no high quality evidence supported insufficient content validity of the LEFS, further assessment of the individual measurement properties was conducted in accordance with the COSMIN methodology for systematic reviews of Patient‐Reported Outcome Measures (PROMs) [26]. Although internal structure and the remaining measurement properties can be assessed, these measurement properties are directly or indirectly related to the content validity of the LEFS. Therefore, their interpretation is strongly dependent on the quality of the content validity of the LEFS. By assessing these measurement properties, a thorough overview of strengths and weaknesses of the LEFS was obtained which can facilitate the further developmen*t* of this frequently used instrument.

### Comparison with the literature

Until now, the literature on the content validity, structural validity, internal consistency, reliability, measurement error, and construct validity (hypotheses testing) of the LEFS in patients with fractures of the lower extremity has not yet been summarized and/or critically appraised using the updated COSMIN criteria. Nonetheless, two previous systematic [24, 39] reviews assessed the reliability, validity, and responsiveness of the LEFS in patients with a range of musculoskeletal disorders. In contrast to our findings, the systematic review of Mehta et al.[24] found the reliability, validity, and responsiveness of the LEFS to be good [24] and rated more than half of the included studies as being of very good to excellent methodological quality. These differences could be explained by differences in the definition of the concept of content validity and other assessment criteria (i.e., MacDermid [40]) instead of using the updated COSMIN guidelines. The study of Shultz et al. [39] did evaluate the responsiveness of the LEFS by using the COSMIN guidelines. However, this study included patients with any condition associated with the lower leg, ankle, or foot, instead of patients with fractures of the lower extremities in particular. They found a lack of consistency for reporting responsiveness among recovery measures used in the lower leg, ankle, or foot studies. Our systematic review results also differ from Morris et al.[25], who assessed outcome measurements following tibia fractures and found the measurement properties of the LEFS to be good. Nevertheless, the authors also stated that if only the fracture patients were considered in the validation studies, all studies would score poorly on the COSMIN checklist, which is in line with the findings of the current review.

### Strengths and limitations

This study included a comprehensive methodological assessment of the LEFS in accordance with the COSMIN methodology for systematic reviews of Patient‐Reported Outcome Measures (PROMs) [26], and thereby rated all properties in the appropriate order (i.e., content validity first), based on well-defined criteria. This study focused on the use of the LEFS patients with fractures of the lower extremity in particular, which differ from patients with other lower extremity dysfunctions. Furthermore, patients with fractures of the lower extremity are a rising source of morbidity associated with a major impact on patients' functional status and health-related quality of life. This is important because measurement properties are context-dependent and have to be evaluated in the context of interest [24]. A possible limitation may be the settings in which the measurement properties of the LEFS were assessed. As only one study [23] included patients that were treated in a primary care setting the generalizability of our findings may be limited for patients that are treated in primary care, such as patients that have sustained a fracture longer ago, or who have a simpler injury.

Another possible limitation may be the small sample sizes of the included studies, in combination of the small amount of the studies we retrieved on the different measurement properties. Although the COSMIN guideline provides the opportunity to pool the results of studies with small sample sizes on several measurement properties (i.e. internal consistency, measurement error, hypothesis testing for construct validity and responsiveness), this is not accounted for in our study as pooling was not feasible [26]. However, in the assessment of the measurement properties content validity and structural validity, we did account for small sample sizes, according to the COSMIN guideline. Another limitation in assessing structural validity was that across the studies included different fit indexes (e.g., CFI, SRMSR, TLI, and RMSEA) were used to report on structural validity, making comparison difficult.

Furthermore, another possible limitation may be the strict inclusion criteria of only including studies, of which at least 75% of the study sample had a lower extremity fracture. This may be why we did not identify additional content validity studies of the LEFS and were not able to include all measurement properties, such as criterion validity and cross-cultural validity. We did consider including studies performed in (slightly) different populations because such studies could provide evidence on the PROM's comprehensibility and (although perhaps to a lesser extent) its relevance and comprehensiveness. However, as our main focus was to investigate the measurement properties of the LEFS in patients with fractures of the lower extremity, instead of all patients with musculoskeletal disorders of the lower extremity, we eventually opted not to do so. Another possible limitation may be our findings' generalizability, as the included studies mostly assessed the LEFS in patients with fractures in the ankle and foot region [23, 31–36]. This could make our systematic review results less generalizable to the whole population of patients with fractures of the lower extremity, such as hip, ankle and/or tibial fractures which form a substantial part of all fractures of the lower extremities. Another point that can be made is the inclusion of studies that assessed the LEFS in four languages, including English [23, 33], Norwegian [32], Chinese [31], and Finnish [34–36]. Nevertheless, no studies assessing cross-cultural validity in patients with fractures of the lower extremities could be identified.

### Implications for practice

In interpreting the scores of the LEFS, one should therefore be aware that not all relevant aspects of physical functioning may be accounted for, such as mobility and self-care. It is not clear if patients find the LEFS comprehensive and perceive the items as relevant and comprehensible. Although the LEFS is often used to assess progress and recovery in treating patients with fractures, no evidence was found to endorse the use of the LEFS in doing so.

### Implications for research

The LEFS needs to be further validated in a well-designed content validity study, which includes a clearly defined construct and involves patients during assessing the different aspects of content validity (i.e., relevance, comprehensiveness, and comprehensibility).

## Conclusion

Although the LEFS is a well-known, frequently used, and easily applicable PROM, there are limitations in the development. This led to an 'inconsistent' rating for content validity of the LEFS, which was supported by very low evidence. Moreover, there is ‘adequate’ evidence that shows that the LEFS has a multidimensional construct, leading to an 'indeterminate’ rating for internal consistency. In interpreting the scores of the LEFS, one should therefore be aware that not all relevant aspects of physical functioning may be accounted for, such as mobility and self-care. For this reason, the LEFS encounters shortcomings regarding its content validity according to the COSMIN guideline [27]. We acknowledge that the LEFS was developed many years before the COSMIN criteria, and the introduction of the dynamic model of health [34], however, we do endorse the fact that PROMS need to be fit for purpose when evaluating current health care. Further validation in a well-designed content validity study is needed, which includes a clearly defined construct and a qualitative part in which not only professionals but also patients with different types of fractures are involved during assessing the different aspects of content validity (i.e., relevance, comprehensiveness, and comprehensibility).

## Data Availability

All data relevant to the study are included in this article or are available as supplementary files.

## Appendix 1

Searchstring PubMed (including Medline)

## LEFS[tiab] OR "lower extremity functional scale"[tiab] OR "lower extremity FS"[tiab] OR "LE functional scale"[tiab] OR "lower extremity scale"[tiab]

Searchstring Embase

## LEFS:ti,ab,kw OR "lower extremity functional scale":ti,ab,kw OR "lower extremity FS":ti,ab,kw OR "LE functional scale":ti,ab,kw OR "lower extremity scale":ti,ab,kw

Searchstring Scopus

## LEFS OR "lower extremity functional scale" OR "lower extremity FS" OR "LE functional scale" OR "lower extremity scale"

Searchstring Cochrane

## References

[1] Donohoe E, Roberts HJ, Miclau T, Kreder H (2020). Management of lower extremity fractures in the elderly: a focus on post-operative rehabilitation. Injury.

[2] Veronese N, Maggi S (2018). Epidemiology and social costs of hip fracture. Injury.

[3] Marks R (2010). Hip fracture epidemiological trends, outcomes, and risk factors, 1970–2009. Int J General Med.

[4] Cheng K, Montgomery S, Housley S, Wheelwright E (2009). Clinical risk factors for hip fracture in young adults under 50 years old. Eur J Trauma Emerg Surg.

[5] Al-Ani AN, Neander G, Samuelsson B, Blomfeldt R, Ekström W, Hedström M (2013). Risk factors for osteoporosis are common in young and middle-aged patients with femoral neck fractures regardless of trauma mechanism. Acta Orthop.

[6] Fredericson M, Jennings F, Beaulieu C, Matheson GO (2006). Stress fractures in athletes. Top Mag Reson Imaging: TMRI.

[7] Sahlin Y (1990). Occurrence of fractures in a defined population: a 1-year study. Injury.

[8] Donaldson LJ, Cook A, Thomson RG (1990). Incidence of fractures in a geographically defined population. J Epidemiol Community Health.

[9] Kaye JA, Jick H (2004). Epidemiology of lower limb fractures in general practice in the United Kingdom. Injury Prevent: J Int Soc Child Adolescent Injury Prevent.

[10] Beerekamp MSH, de Muinck Keizer RJO, Schep NWL, Ubbink DT, Panneman MJM, Goslings JC (2017). Epidemiology of extremity fractures in the Netherlands. Injury.

[11] van Staa TP, Dennison EM, Leufkens HG, Cooper C (2001). Epidemiology of fractures in England and Wales. Bone.

[12] MacKenzie EJ, Bosse MJ, Pollak AN, Webb LX, Swiontkowski MF, Kellam JF (2005). Long-term persistence of disability following severe lower-limb trauma. Results of a seven-year follow-up. J Bone Joint Surg Am.

[13] Miclau T, Van Lieshout EMM (2020). Optimizing patient function after musculoskeletal trauma: an introduction. Injury.

[14] Dawson J, Doll H, Fitzpatrick R, Jenkinson C, Carr AJ. The routine use of patient reported outcome measures in healthcare settings. BMJ (Clinical Research ed). 2010;340:c186. 10.1136/bmj.c186 20083546

[15] Sepehri A, Slobogean GP (2020). Which study outcomes change practice. Injury.

[16] Lübbeke A (2018). Research methodology for orthopaedic surgeons, with a focus on outcome. EFORT Open Rev.

[17] Slevin ML, Plant H, Lynch D, Drinkwater J, Gregory WM (1988). Who should measure quality of life, the doctor or the patient?. Br J Cancer.

[18] de Munter L, Polinder S, van de Ree CLP, Kruithof N, Lansink KWW, Steyerberg EW (2019). Predicting health status in the first year after trauma. Br J Surg.

[19] Celso B, Tepas J, Langland-Orban B, Pracht E, Papa L, Lottenberg L, et al. A systematic review and meta-analysis comparing outcome of severely injured patients treated in trauma centers following the establishment of trauma systems. J Trauma. 2006;60(2):371–8; discussion 8. 10.1097/01.ta.0000197916.99629.eb 16508498

[20] Van Lieshout EMM, Wijffels MME (2020). Patient-reported outcomes: Which ones are most relevant?. Injury.

[21] Patient-Reported Outcomes [Available from: https://www.qualityforum.org/Projects/n-r/Patient-Reported_Outcomes/Patient-Reported_Outcomes.aspx.

[22] Higgins JPT TJ, Chandler J, Cumpston M, Li T, Page MJ, Welch VA Cochrane Handbook for Systematic Reviews of Interventions version 6.0 (updated July 2019). In: Higgins JPT TJ, Chandler J, Cumpston M, Li T, Page MJ, Welch VA editor.: Wiley; 2019.

[23] Binkley JM, Stratford PW, Lott SA, Riddle DL (1999). The Lower Extremity Functional Scale (LEFS): scale development, measurement properties, and clinical application. North American Orthopaedic Rehabilitation Research Network. Phys Therapy..

[24] Mehta SP, Fulton A, Quach C, Thistle M, Toledo C, Evans NA (2016). Measurement Properties of the Lower Extremity Functional Scale: A Systematic Review. J Orthop Sports Phys Ther.

[25] Morris R, Pallister I, Trickett RW (2019). Measuring outcomes following tibial fracture. Injury.

[26] Prinsen CAC, Mokkink LB, Bouter LM, Alonso J, Patrick DL, de Vet HCW (2018). COSMIN guideline for systematic reviews of patient-reported outcome measures. Quality Life Res.

[27] Terwee CB, Prinsen CAC, Chiarotto A, Westerman MJ, Patrick DL, Alonso J (2018). COSMIN methodology for evaluating the content validity of patient-reported outcome measures: a Delphi study. Quality Life Res.

[28] Prinsen CA, Vohra S, Rose MR, Boers M, Tugwell P, Clarke M (2016). How to select outcome measurement instruments for outcomes included in a "Core Outcome Set" - a practical guideline. Trials.

[29] Mokkink LB, Terwee CB, Patrick DL, Alonso J, Stratford PW, Knol DL (2010). The COSMIN study reached international consensus on taxonomy, terminology, and definitions of measurement properties for health-related patient-reported outcomes. J Clin Epidemiol.

[30] Pinsker E, Daniels TR, Inrig T, Warmington K, Beaton DE (2013). The ability of outcome questionnaires to capture patient concerns following ankle reconstruction. Foot Ankle Int.

[31] Hsu CY, Tsai YS, Yau CS, Shie HH, Wu CM (2019). Differences in gait and trunk movement between patients after ankle fracture and healthy subjects. Biomed Eng Online.

[32] Garratt AM, Naumann MG, Sigurdsen U, Utvåg SE, Stavem K (2018). Evaluation of three patient reported outcome measures following operative fixation of closed ankle fractures. BMC Musculoskelet Disord.

[33] Lin CW, Moseley AM, Refshauge KM, Bundy AC (2009). The lower extremity functional scale has good clinimetric properties in people with ankle fracture. Phys Ther.

[34] Repo JP, Tukiainen EJ, Roine RP, Ilves O, Järvenpää S, Häkkinen A (2017). Reliability and validity of the Finnish version of the Lower Extremity Functional Scale (LEFS). Disabil Rehabil.

[35] Repo JP, Tukiainen EJ, Roine RP, Sampo M, Elin H, Häkkinen AH (2019). Rasch analysis of the Lower Extremity Functional Scale for foot and ankle patients. Disabil Rehabil.

[36] Ponkilainen VT, Tukiainen EJ, Uimonen MM, Häkkinen AH, Repo JP (2020). Assessment of the structural validity of three foot and ankle specific patient-reported outcome measures. Foot Ankle Surg.

[37] McDowell I, Spasoff RA, Kristjansson B (2004). On the classification of population health measurements. Am J Public Health.

[38] Organization WH. Health promotion : a discussion document on the concept and principles : summary report of the Working Group on Concept and Principles of Health Promotion Copenhagen: WHO Regional Office for Europe1984 [Available from: https://apps.who.int/iris/handle/10665/107835.

[39] Shultz S, Olszewski A, Ramsey O, Schmitz M, Wyatt V, Cook C (2013). A systematic review of outcome tools used to measure lower leg conditions. Int J Sports Phys Ther.

[40] Roy JS, MacDermid JC, Woodhouse LJ (2009). Measuring shoulder function: a systematic review of four questionnaires. Arthritis Rheum.

